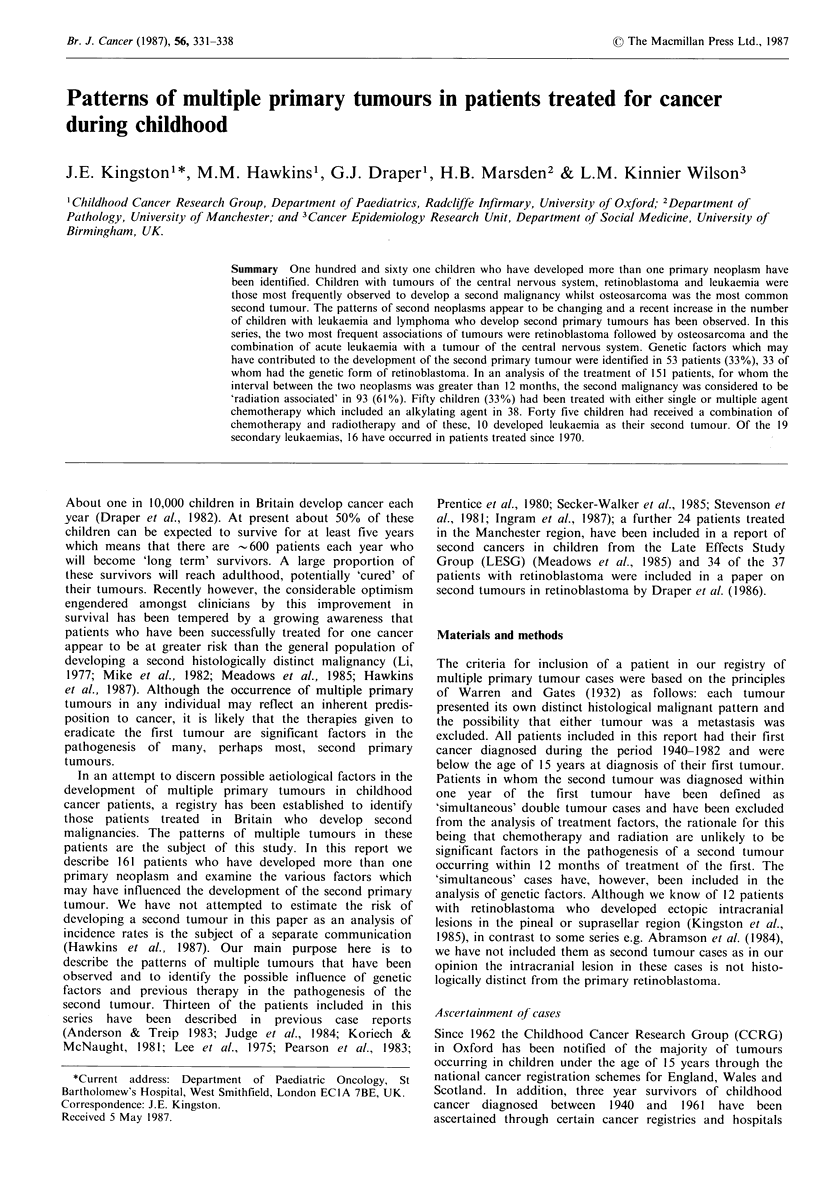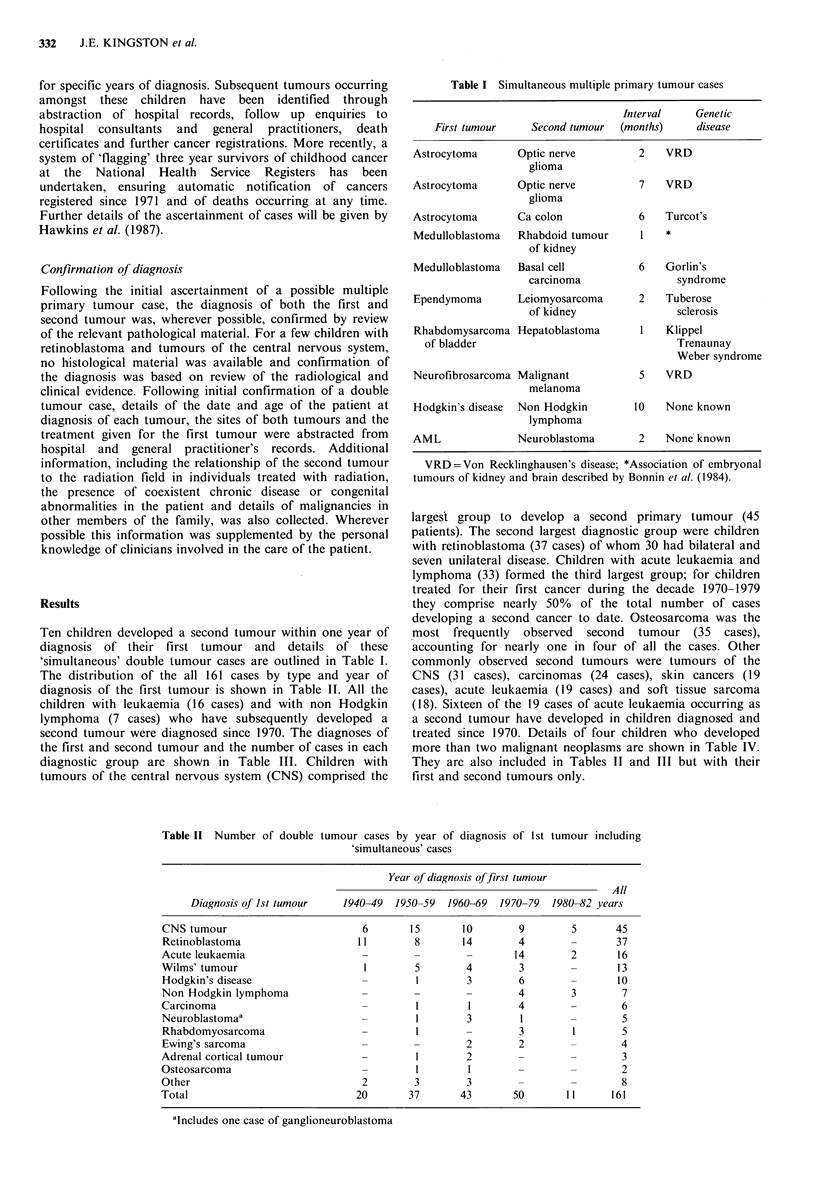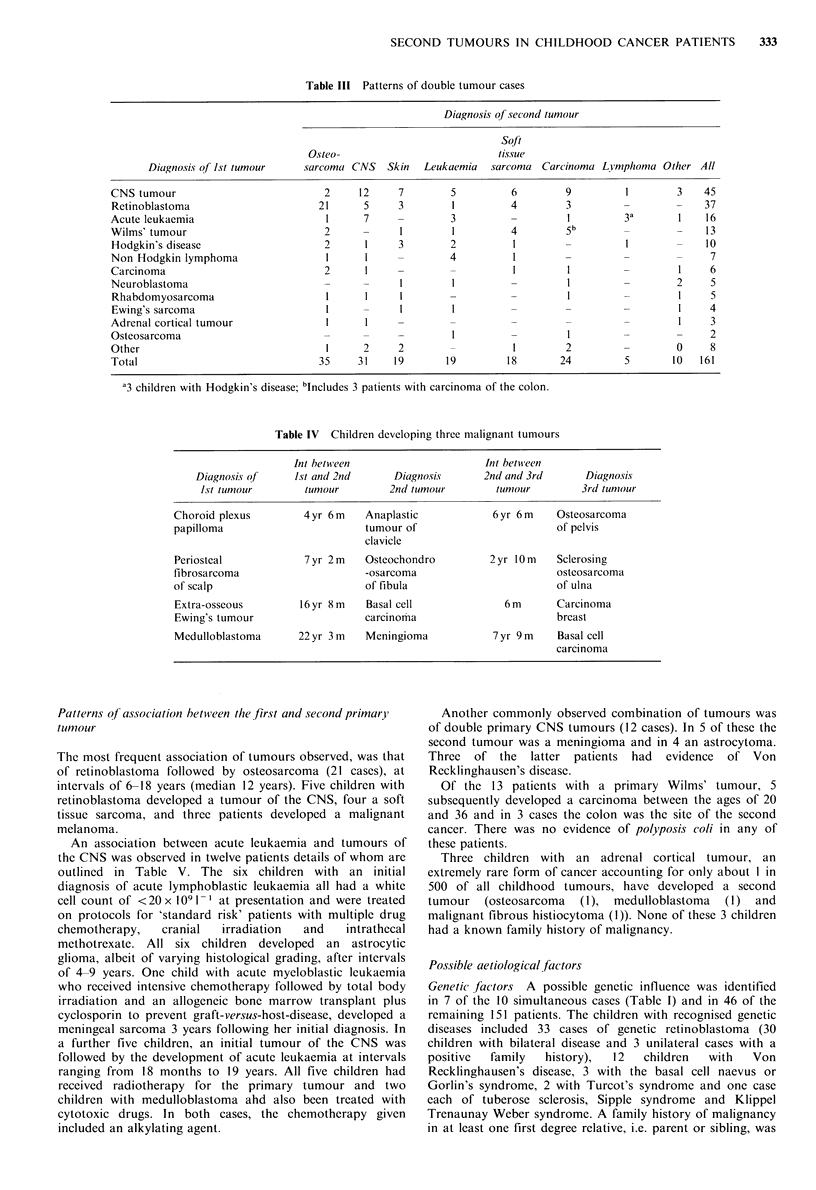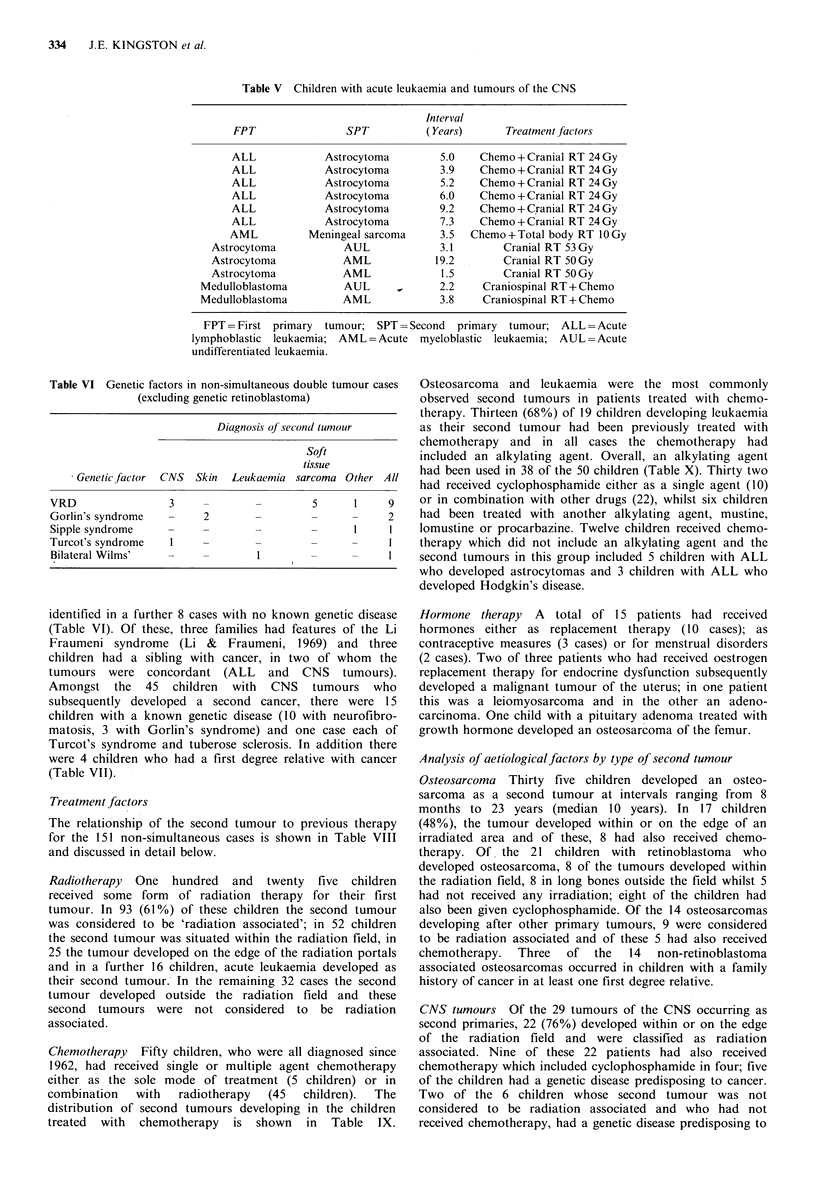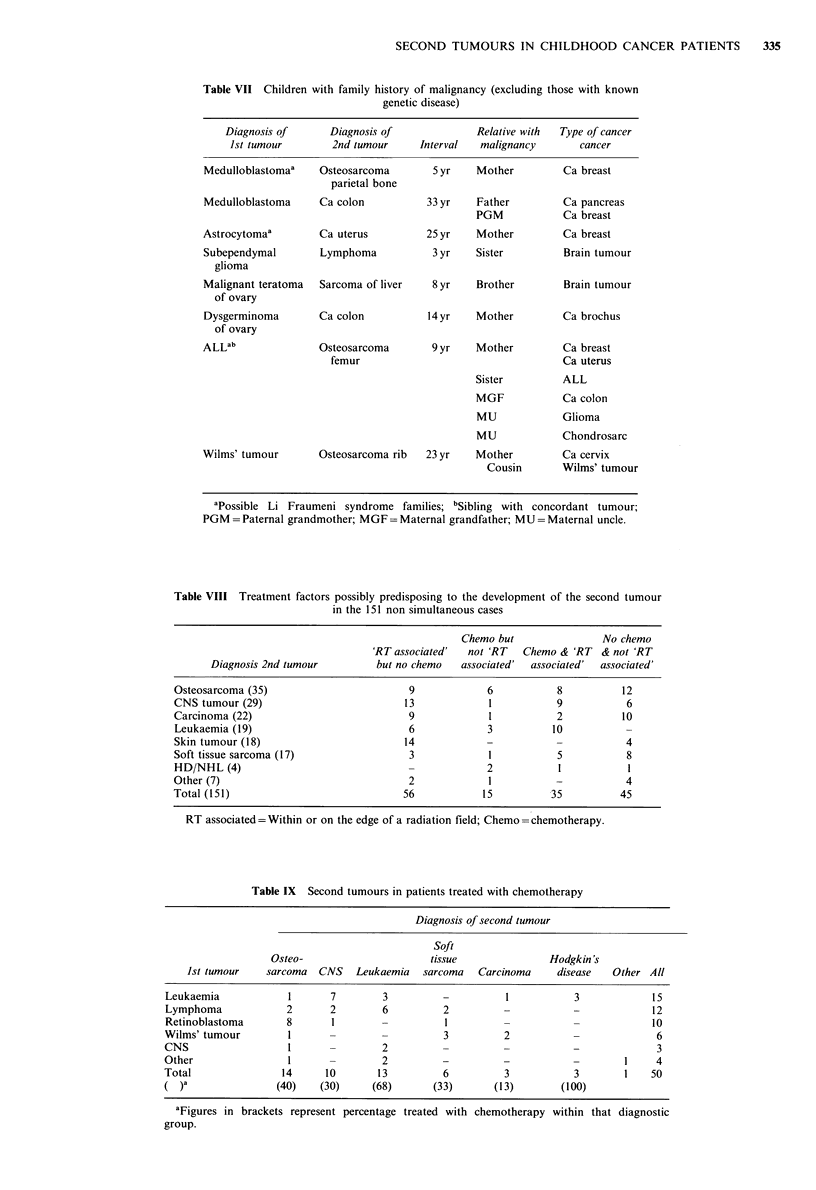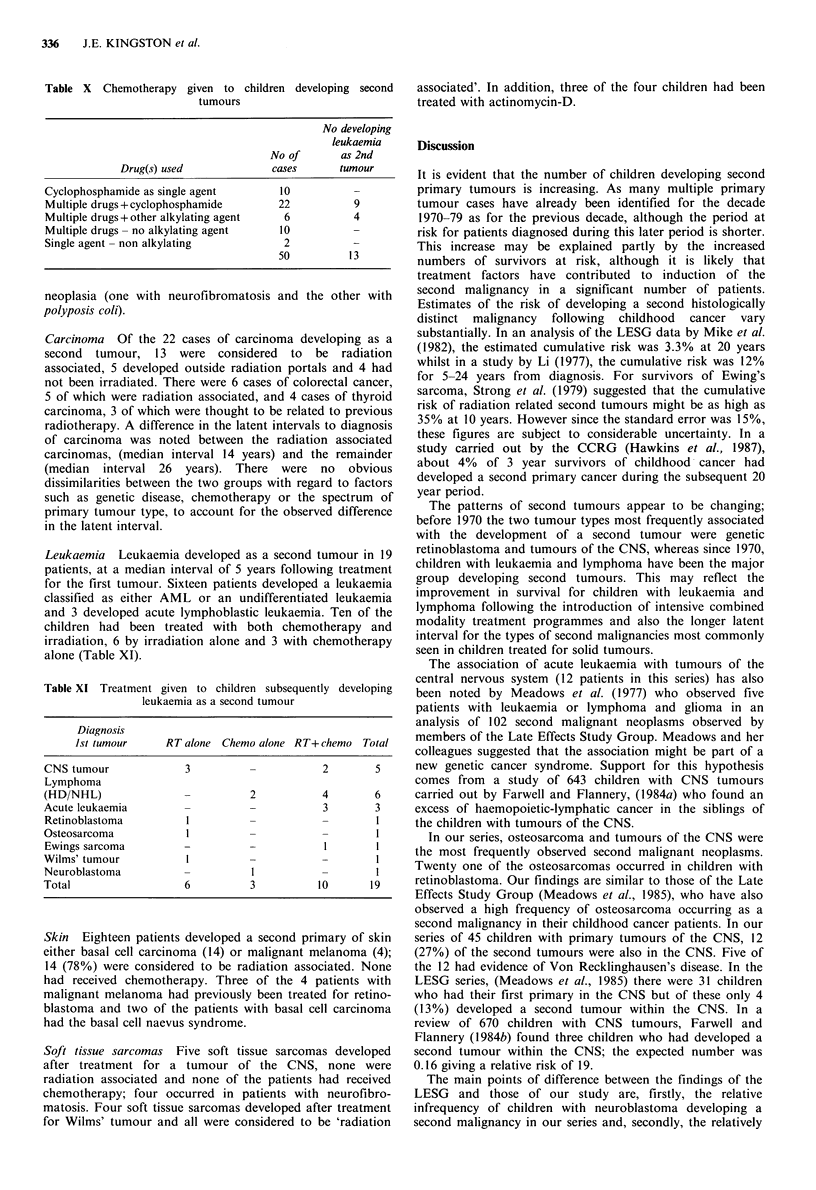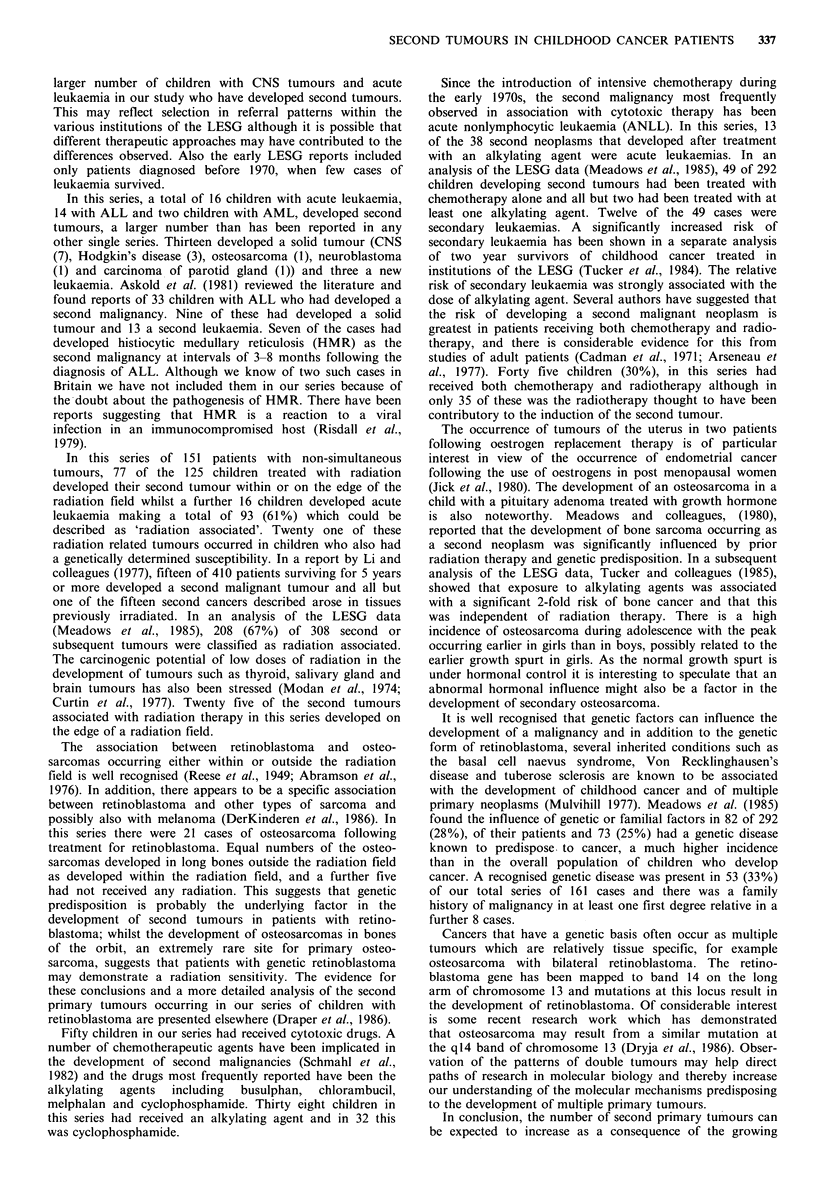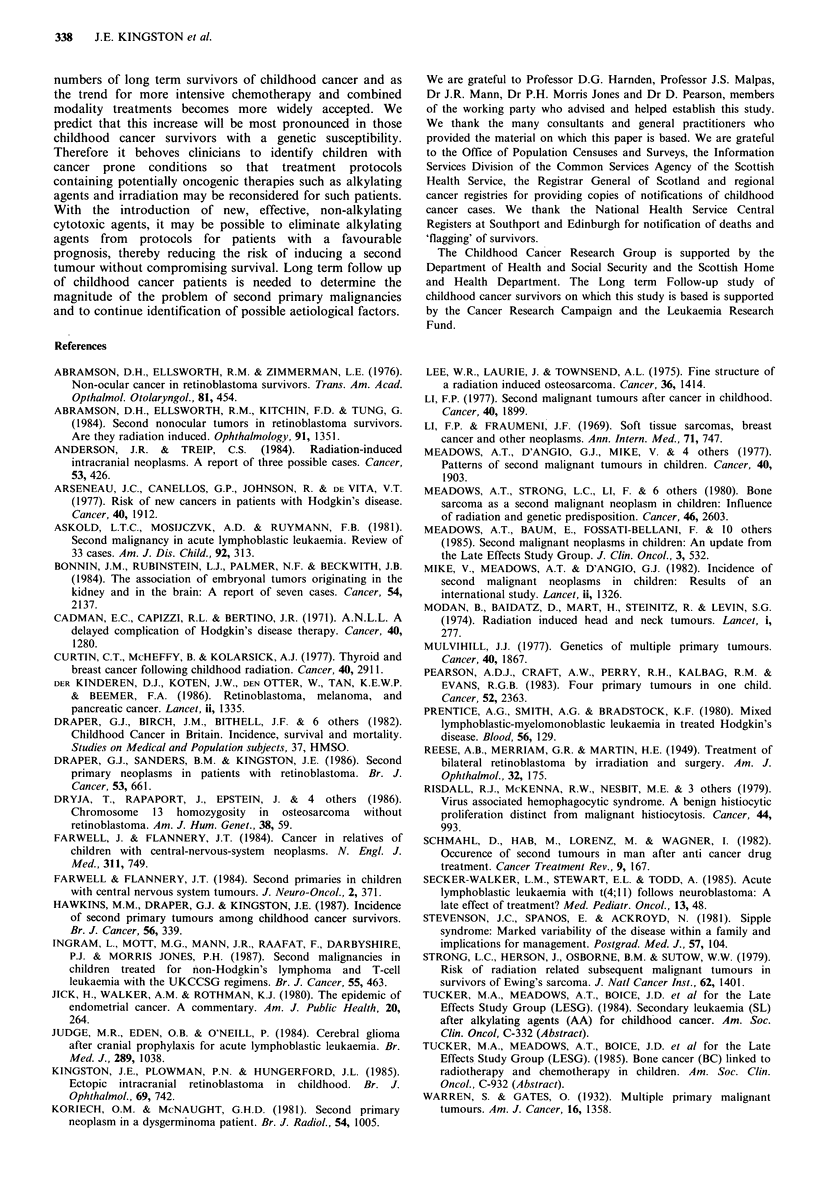# Patterns of multiple primary tumours in patients treated for cancer during childhood.

**DOI:** 10.1038/bjc.1987.199

**Published:** 1987-09

**Authors:** J. E. Kingston, M. M. Hawkins, G. J. Draper, H. B. Marsden, L. M. Kinnier Wilson

**Affiliations:** Department of Paediatrics, Radcliffe Infirmary, University of Oxford, UK.

## Abstract

One hundred and sixty one children who have developed more than one primary neoplasm have been identified. Children with tumours of the central nervous system, retinoblastoma and leukaemia were those most frequently observed to develop a second malignancy whilst osteosarcoma was the most common second tumour. The patterns of second neoplasms appear to be changing and a recent increase in the number of children with leukaemia and lymphoma who develop second primary tumours has been observed. In this series, the two most frequent associations of tumours were retinoblastoma followed by osteosarcoma and the combination of acute leukaemia with a tumour of the central nervous system. Genetic factors which may have contributed to the development of the second primary tumour were identified in 53 patients (33%), 33 of whom had the genetic form of retinoblastoma. In an analysis of the treatment of 151 patients, for whom the interval between the two neoplasms was greater than 12 months, the second malignancy was considered to be 'radiation associated' in 93 (61%). Fifty children (33%) had been treated with either single or multiple agent chemotherapy which included an alkylating agent in 38. Forty five children had received a combination of chemotherapy and radiotherapy and of these, 10 developed leukaemia as their second tumour. Of the 19 secondary leukaemias, 16 have occurred in patients treated since 1970.


*Br. J. Cancer* (1987), **56**, 331–338 © The Macmillan Press Ltd., 1987

### Patterns of multiple primary tumours in patients treated for cancer during childhood

J.E. Kingston[1]*, M.M. Hawkins[1], G.J. Draper[1], H.B. Marsden[2] & L.M. Kinnier Wilson[3]

[1]*Childhood Cancer Research Group, Department of Paediatrics, Radcliffe Infirmary, University of Oxford;* [2]*Department of Pathology, University of Manchester; and* [3]*Cancer Epidemiology Research Unit, Department of Social Medicine, University of Birmingham, UK.*

**Summary** One hundred and sixty one children who have developed more than one primary neoplasm have been identified. Children with tumours of the central nervous system, retinoblastoma and leukaemia were those most frequently observed to develop a second malignancy whilst osteosarcoma was the most common second tumour. The patterns of second neoplasms appear to be changing and a recent increase in the number of children with leukaemia and lymphoma who develop second primary tumours has been observed. In this series, the two most frequent associations of tumours were retinoblastoma followed by osteosarcoma and the combination of acute leukaemia with a tumour of the central nervous system. Genetic factors which may have contributed to the development of the second primary tumour were identified in 53 patients (33%), 33 of whom had the genetic form of retinoblastoma. In an analysis of the treatment of 151 patients, for whom the interval between the two neoplasms was greater than 12 months, the second malignancy was considered to be 'radiation associated' in 93 (61%). Fifty children (33%) had been treated with either single or multiple agent chemotherapy which included an alkylating agent in 38. Forty five children had received a combination of chemotherapy and radiotherapy and of these, 10 developed leukaemia as their second tumour. Of the 19 secondary leukaemias, 16 have occurred in patients treated since 1970.

About one in 10,000 children in Britain develop cancer each year (Draper *et al.*, 1982). At present about 50% of these children can be expected to survive for at least five years which means that there are ~600 patients each year who will become 'long term' survivors. A large proportion of these survivors will reach adulthood, potentially 'cured' of their tumours. Recently however, the considerable optimism engendered amongst clinicians by this improvement in survival has been tempered by a growing awareness that patients who have been successfully treated for one cancer appear to be at greater risk than the general population of developing a second histologically distinct malignancy (Li, 1977; Mike *et al.*, 1982; Meadows *et al.*, 1985; Hawkins *et al.*, 1987). Although the occurrence of multiple primary tumours in any individual may reflect an inherent predisposition to cancer, it is likely that the therapies given to eradicate the first tumour are significant factors in the pathogenesis of many, perhaps most, second primary tumours.

In an attempt to discern possible aetiological factors in the development of multiple primary tumours in childhood cancer patients, a registry has been established to identify those patients treated in Britain who develop second malignancies. The patterns of multiple tumours in these patients are the subject of this study. In this report we describe 161 patients who have developed more than one primary neoplasm and examine the various factors which may have influenced the development of the second primary tumour. We have not attempted to estimate the risk of developing a second tumour in this paper as an analysis of incidence rates is the subject of a separate communication (Hawkins *et al.*, 1987). Our main purpose here is to describe the patterns of multiple tumours that have been observed and to identify the possible influence of genetic factors and previous therapy in the pathogenesis of the second tumour. Thirteen of the patients included in this series have been described in previous case reports (Anderson & Treip 1983; Judge *et al.*, 1984; Koriech & McNaught, 1981; Lee *et al.*, 1975; Pearson *et al.*, 1983; Prentice *et al.*, 1980; Secker-Walker *et al.*, 1985; Stevenson *et al.*, 1981; Ingram *et al.*, 1987); a further 24 patients treated in the Manchester region, have been included in a report of second cancers in children from the Late Effects Study Group (LESG) (Meadows *et al.*, 1985) and 34 of the 37 patients with retinoblastoma were included in a paper on second tumours in retinoblastoma by Draper *et al.* (1986).

*Current address: Department of Paediatric Oncology, St Bartholomew's Hospital, West Smithfield, London EC1A 7BE, UK.
Correspondence: J.E. Kingston.
Received 5 May 1987.

### Materials and methods

The criteria for inclusion of a patient in our registry of multiple primary tumour cases were based on the principles of Warren and Gates (1932) as follows: each tumour presented its own distinct histological malignant pattern and the possibility that either tumour was a metastasis was excluded. All patients included in this report had their first cancer diagnosed during the period 1940–1982 and were below the age of 15 years at diagnosis of their first tumour. Patients in whom the second tumour was diagnosed within one year of the first tumour have been defined as 'simultaneous' double tumour cases and have been excluded from the analysis of treatment factors, the rationale for this being that chemotherapy and radiation are unlikely to be significant factors in the pathogenesis of a second tumour occurring within 12 months of treatment of the first. The 'simultaneous' cases have, however, been included in the analysis of genetic factors. Although we know of 12 patients with retinoblastoma who developed ectopic intracranial lesions in the pineal or suprasellar region (Kingston *et al.*, 1985), in contrast to some series e.g. Abramson *et al.* (1984), we have not included them as second tumour cases as in our opinion the intracranial lesion in these cases is not histologically distinct from the primary retinoblastoma.

### *Ascertainment of cases*

Since 1962 the Childhood Cancer Research Group (CCRG) in Oxford has been notified of the majority of tumours occurring in children under the age of 15 years through the national cancer registration schemes for England, Wales and Scotland. In addition, three year survivors of childhood cancer diagnosed between 1940 and 1961 have been ascertained through certain cancer registries and hospitals for specific years of diagnosis. Subsequent tumours occurring amongst these children have been identified through abstraction of hospital records, follow up enquiries to hospital consultants and general practitioners, death certificates and further cancer registrations. More recently, a system of 'flagging' three year survivors of childhood cancer at the National Health Service Registers has been undertaken, ensuring automatic notification of cancers registered since 1971 and of deaths occurring at any time. Further details of the ascertainment of cases will be given by Hawkins *et al.* (1987).

*Confirmation of diagnosis*

Following the initial ascertainment of a possible multiple primary tumour case, the diagnosis of both the first and second tumour was, wherever possible, confirmed by review of the relevant pathological material. For a few children with retinoblastoma and tumours of the central nervous system, no histological material was available and confirmation of the diagnosis was based on review of the radiological and clinical evidence. Following initial confirmation of a double tumour case, details of the date and age of the patient at diagnosis of each tumour, the sites of both tumours and the treatment given for the first tumour were abstracted from hospital and general practitioner's records. Additional information, including the relationship of the second tumour to the radiation field in individuals treated with radiation, the presence of coexistent chronic disease or congenital abnormalities in the patient and details of malignancies in other members of the family, was also collected. Wherever possible this information was supplemented by the personal knowledge of clinicians involved in the care of the patient.

### Results

Ten children developed a second tumour within one year of diagnosis of their first tumour and details of these 'simultaneous' double tumour cases are outlined in Table I. The distribution of the all 161 cases by type and year of diagnosis of the first tumour is shown in Table II. All the children with leukaemia (16 cases) and with non Hodgkin lymphoma (7 cases) who have subsequently developed a second tumour were diagnosed since 1970. The diagnoses of the first and second tumour and the number of cases in each diagnostic group are shown in Table III. Children with tumours of the central nervous system (CNS) comprised the largest group to develop a second primary tumour (45 patients). The second largest diagnostic group were children with retinoblastoma (37 cases) of whom 30 had bilateral and seven unilateral disease. Children with acute leukaemia and lymphoma (33) formed the third largest group; for children treated for their first cancer during the decade 1970–1979 they comprise nearly 50% of the total number of cases developing a second cancer to date. Osteosarcoma was the most frequently observed second tumour (35 cases), accounting for nearly one in four of all the cases. Other commonly observed second tumours were tumours of the CNS (31 cases), carcinomas (24 cases), skin cancers (19 cases), acute leukaemia (19 cases) and soft tissue sarcoma (18). Sixteen of the 19 cases of acute leukaemia occurring as a second tumour have developed in children diagnosed and treated since 1970. Details of four children who developed more than two malignant neoplasms are shown in Table IV. They are also included in Tables II and III but with their first and second tumours only.

**Table I** Simultaneous multiple primary tumour cases

| *First tumour* | *Second tumour* | *Interval (months)* | *Genetic disease* |
|---|---|---|---|
| Astrocytoma | Optic nerve glioma | 2 | VRD |
| Astrocytoma | Optic nerve glioma | 7 | VRD |
| Astrocytoma | Ca colon | 6 | Turcot's |
| Medulloblastoma | Rhabdoid tumour of kidney | 1 | * |
| Medulloblastoma | Basal cell carcinoma | 6 | Gorlin's syndrome |
| Ependymoma | Leiomyosarcoma of kidney | 2 | Tuberose sclerosis |
| Rhabdomysarcoma of bladder | Hepatoblastoma | 1 | Klippel Trenaunay Weber syndrome |
| Neurofibrosarcoma | Malignant melanoma | 5 | VRD |
| Hodgkin's disease | Non Hodgkin lymphoma | 10 | None known |
| AML | Neuroblastoma | 2 | None known |

VRD = Von Recklinghausen's disease; *Association of embryonal tumours of kidney and brain described by Bonnin *et al.* (1984).

**Table II** Number of double tumour cases by year of diagnosis of 1st tumour including 'simultaneous' cases

| *Diagnosis of 1st tumour* | *Year of diagnosis of first tumour* 1940–49 | 1950–59 | 1960–69 | 1970–79 | 1980–82 | *All years* |
|---|---|---|---|---|---|---|
| CNS tumour | 6 | 15 | 10 | 9 | 5 | 45 |
| Retinoblastoma | 11 | 8 | 14 | 4 | – | 37 |
| Acute leukaemia | – | – | – | 14 | 2 | 16 |
| Wilms' tumour | 1 | 5 | 4 | 3 | – | 13 |
| Hodgkin's disease | – | 1 | 3 | 6 | – | 10 |
| Non Hodgkin lymphoma | – | – | – | 4 | 3 | 7 |
| Carcinoma | – | 1 | 1 | 4 | – | 6 |
| Neuroblastoma[a] | – | 1 | 3 | 1 | – | 5 |
| Rhabdomyosarcoma | – | 1 | – | 3 | 1 | 5 |
| Ewing's sarcoma | – | – | 2 | 2 | – | 4 |
| Adrenal cortical tumour | – | 1 | 2 | – | – | 3 |
| Osteosarcoma | – | 1 | 1 | – | – | 2 |
| Other | 2 | 3 | 3 | – | – | 8 |
| Total | 20 | 37 | 43 | 50 | 11 | 161 |

[a]Includes one case of ganglioneuroblastoma

**Table III** Patterns of double tumour cases

| | Diagnosis of second tumour | | | | | | | | |
|---|---|---|---|---|---|---|---|---|---|
| *Diagnosis of 1st tumour* | *Osteo-sarcoma* | *CNS* | *Skin* | *Leukaemia* | *Soft tissue sarcoma* | *Carcinoma* | *Lymphoma* | *Other* | *All* |
| CNS tumour | 2 | 12 | 7 | 5 | 6 | 9 | 1 | 3 | 45 |
| Retinoblastoma | 21 | 5 | 3 | 1 | 4 | 3 | – | – | 37 |
| Acute leukaemia | 1 | 7 | – | 3 | – | 1 | 3[a] | 1 | 16 |
| Wilms' tumour | 2 | – | 1 | 1 | 4 | 5[b] | – | – | 13 |
| Hodgkin's disease | 2 | 1 | 3 | 2 | 1 | – | 1 | – | 10 |
| Non Hodgkin lymphoma | 1 | 1 | – | 4 | 1 | – | – | – | 7 |
| Carcinoma | 2 | 1 | – | – | 1 | 1 | – | 1 | 6 |
| Neuroblastoma | – | – | 1 | 1 | – | 1 | – | 2 | 5 |
| Rhabdomyosarcoma | 1 | 1 | 1 | – | – | 1 | – | 1 | 5 |
| Ewing's sarcoma | 1 | – | 1 | 1 | – | – | – | 1 | 4 |
| Adrenal cortical tumour | 1 | 1 | – | – | – | – | – | 1 | 3 |
| Osteosarcoma | – | – | – | 1 | – | 1 | – | – | 2 |
| Other | 1 | 2 | 2 | – | 1 | 2 | – | 0 | 8 |
| Total | 35 | 31 | 19 | 19 | 18 | 24 | 5 | 10 | 161 |

[a]3 children with Hodgkin's disease; [b]Includes 3 patients with carcinoma of the colon.

**Table IV** Children developing three malignant tumours

| *Diagnosis of 1st tumour* | *Int between 1st and 2nd tumour* | *Diagnosis 2nd tumour* | *Int between 2nd and 3rd tumour* | *Diagnosis 3rd tumour* |
|---|---|---|---|---|
| Choroid plexus papilloma | 4 yr 6 m | Anaplastic tumour of clavicle | 6 yr 6 m | Osteosarcoma of pelvis |
| Periosteal fibrosarcoma of scalp | 7 yr 2 m | Osteochondro -osarcoma of fibula | 2 yr 10 m | Sclerosing osteosarcoma of ulna |
| Extra-osseous Ewing's tumour | 16 yr 8 m | Basal cell carcinoma | 6 m | Carcinoma breast |
| Medulloblastoma | 22 yr 3 m | Meningioma | 7 yr 9 m | Basal cell carcinoma |

*Patterns of association between the first and second primary tumour*

The most frequent association of tumours observed, was that of retinoblastoma followed by osteosarcoma (21 cases), at intervals of 6–18 years (median 12 years). Five children with retinoblastoma developed a tumour of the CNS, four a soft tissue sarcoma, and three patients developed a malignant melanoma.

An association between acute leukaemia and tumours of the CNS was observed in twelve patients details of whom are outlined in Table V. The six children with an initial diagnosis of acute lymphoblastic leukaemia all had a white cell count of $<20 \times 10^9 l^{-1}$ at presentation and were treated on protocols for 'standard risk' patients with multiple drug chemotherapy, cranial irradiation and intrathecal methotrexate. All six children developed an astrocytic glioma, albeit of varying histological grading, after intervals of 4–9 years. One child with acute myeloblastic leukaemia who received intensive chemotherapy followed by total body irradiation and an allogeneic bone marrow transplant plus cyclosporin to prevent graft-*versus*-host-disease, developed a meningeal sarcoma 3 years following her initial diagnosis. In a further five children, an initial tumour of the CNS was followed by the development of acute leukaemia at intervals ranging from 18 months to 19 years. All five children had received radiotherapy for the primary tumour and two children with medulloblastoma ahd also been treated with cytotoxic drugs. In both cases, the chemotherapy given included an alkylating agent.

Another commonly observed combination of tumours was of double primary CNS tumours (12 cases). In 5 of these the second tumour was a meningioma and in 4 an astrocytoma. Three of the latter patients had evidence of Von Recklinghausen's disease.

Of the 13 patients with a primary Wilms' tumour, 5 subsequently developed a carcinoma between the ages of 20 and 36 and in 3 cases the colon was the site of the second cancer. There was no evidence of *polyposis coli* in any of these patients.

Three children with an adrenal cortical tumour, an extremely rare form of cancer accounting for only about 1 in 500 of all childhood tumours, have developed a second tumour (osteosarcoma (1), medulloblastoma (1) and malignant fibrous histiocytoma (1)). None of these 3 children had a known family history of malignancy.

*Possible aetiological factors*

*Genetic factors* A possible genetic influence was identified in 7 of the 10 simultaneous cases (Table I) and in 46 of the remaining 151 patients. The children with recognised genetic diseases included 33 cases of genetic retinoblastoma (30 children with bilateral disease and 3 unilateral cases with a positive family history), 12 children with Von Recklinghausen's disease, 3 with the basal cell naevus or Gorlin's syndrome, 2 with Turcot's syndrome and one case each of tuberose sclerosis, Sipple syndrome and Klippel Trenaunay Weber syndrome. A family history of malignancy in at least one first degree relative, i.e. parent or sibling, was

**Table V** Children with acute leukaemia and tumours of the CNS

| *FPT* | *SPT* | *Interval (Years)* | *Treatment factors* |
|---|---|---|---|
| ALL | Astrocytoma | 5.0 | Chemo + Cranial RT 24 Gy |
| ALL | Astrocytoma | 3.9 | Chemo + Cranial RT 24 Gy |
| ALL | Astrocytoma | 5.2 | Chemo + Cranial RT 24 Gy |
| ALL | Astrocytoma | 6.0 | Chemo + Cranial RT 24 Gy |
| ALL | Astrocytoma | 9.2 | Chemo + Cranial RT 24 Gy |
| ALL | Astrocytoma | 7.3 | Chemo + Cranial RT 24 Gy |
| AML | Meningeal sarcoma | 3.5 | Chemo + Total body RT 10 Gy |
| Astrocytoma | AUL | 3.1 | Cranial RT 53 Gy |
| Astrocytoma | AML | 19.2 | Cranial RT 50 Gy |
| Astrocytoma | AML | 1.5 | Cranial RT 50 Gy |
| Medulloblastoma | AUL | 2.2 | Craniospinal RT + Chemo |
| Medulloblastoma | AML | 3.8 | Craniospinal RT + Chemo |

FPT = First primary tumour; SPT = Second primary tumour; ALL = Acute lymphoblastic leukaemia; AML = Acute myeloblastic leukaemia; AUL = Acute undifferentiated leukaemia.

**Table VI** Genetic factors in non-simultaneous double tumour cases (excluding genetic retinoblastoma)

| | *Diagnosis of second tumour* | | | | | |
|---|---|---|---|---|---|---|
| *Genetic factor* | *CNS* | *Skin* | *Leukaemia* | *Soft tissue sarcoma* | *Other* | *All* |
| VRD | 3 | – | – | 5 | 1 | 9 |
| Gorlin's syndrome | – | 2 | – | – | – | 2 |
| Sipple syndrome | – | – | – | – | 1 | 1 |
| Turcot's syndrome | 1 | – | – | – | – | 1 |
| Bilateral Wilms' | – | – | 1 | – | – | 1 |

identified in a further 8 cases with no known genetic disease (Table VI). Of these, three families had features of the Li Fraumeni syndrome (Li & Fraumeni, 1969) and three children had a sibling with cancer, in two of whom the tumours were concordant (ALL and CNS tumours). Amongst the 45 children with CNS tumours who subsequently developed a second cancer, there were 15 children with a known genetic disease (10 with neurofibromatosis, 3 with Gorlin's syndrome) and one case each of Turcot's syndrome and tuberose sclerosis. In addition there were 4 children who had a first degree relative with cancer (Table VII).

*Treatment factors*

The relationship of the second tumour to previous therapy for the 151 non-simultaneous cases is shown in Table VIII and discussed in detail below.

*Radiotherapy* One hundred and twenty five children received some form of radiation therapy for their first tumour. In 93 (61%) of these children the second tumour was considered to be 'radiation associated'; in 52 children the second tumour was situated within the radiation field, in 25 the tumour developed on the edge of the radiation portals and in a further 16 children, acute leukaemia developed as their second tumour. In the remaining 32 cases the second tumour developed outside the radiation field and these second tumours were not considered to be radiation associated.

*Chemotherapy* Fifty children, who were all diagnosed since 1962, had received single or multiple agent chemotherapy either as the sole mode of treatment (5 children) or in combination with radiotherapy (45 children). The distribution of second tumours developing in the children treated with chemotherapy is shown in Table IX. Osteosarcoma and leukaemia were the most commonly observed second tumours in patients treated with chemotherapy. Thirteen (68%) of 19 children developing leukaemia as their second tumour had been previously treated with chemotherapy and in all cases the chemotherapy had included an alkylating agent. Overall, an alkylating agent had been used in 38 of the 50 children (Table X). Thirty two had received cyclophosphamide either as a single agent (10) or in combination with other drugs (22), whilst six children had been treated with another alkylating agent, mustine, lomustine or procarbazine. Twelve children received chemotherapy which did not include an alkylating agent and the second tumours in this group included 5 children with ALL who developed astrocytomas and 3 children with ALL who developed Hodgkin's disease.

*Hormone therapy* A total of 15 patients had received hormones either as replacement therapy (10 cases); as contraceptive measures (3 cases) or for menstrual disorders (2 cases). Two of three patients who had received oestrogen replacement therapy for endocrine dysfunction subsequently developed a malignant tumour of the uterus; in one patient this was a leiomyosarcoma and in the other an adenocarcinoma. One child with a pituitary adenoma treated with growth hormone developed an osteosarcoma of the femur.

*Analysis of aetiological factors by type of second tumour*

*Osteosarcoma* Thirty five children developed an osteosarcoma as a second tumour at intervals ranging from 8 months to 23 years (median 10 years). In 17 children (48%), the tumour developed within or on the edge of an irradiated area and of these, 8 had also received chemotherapy. Of the 21 children with retinoblastoma who developed osteosarcoma, 8 of the tumours developed within the radiation field, 8 in long bones outside the field whilst 5 had not received any irradiation; eight of the children had also been given cyclophosphamide. Of the 14 osteosarcomas developing after other primary tumours, 9 were considered to be radiation associated and of these 5 had also received chemotherapy. Three of the 14 non-retinoblastoma associated osteosarcomas occurred in children with a family history of cancer in at least one first degree relative.

*CNS tumours* Of the 29 tumours of the CNS occurring as second primaries, 22 (76%) developed within or on the edge of the radiation field and were classified as radiation associated. Nine of these 22 patients had also received chemotherapy which included cyclophosphamide in four; five of the children had a genetic disease predisposing to cancer. Two of the 6 children whose second tumour was not considered to be radiation associated and who had not received chemotherapy, had a genetic disease predisposing to

**Table VII** Children with family history of malignancy (excluding those with known genetic disease)

| *Diagnosis of 1st tumour* | *Diagnosis of 2nd tumour* | *Interval* | *Relative with malignancy* | *Type of cancer cancer* |
|---|---|---|---|---|
| Medulloblastoma[a] | Osteosarcoma parietal bone | 5 yr | Mother | Ca breast |
| Medulloblastoma | Ca colon | 33 yr | Father<br>PGM | Ca pancreas<br>Ca breast |
| Astrocytoma[a] | Ca uterus | 25 yr | Mother | Ca breast |
| Subependymal glioma | Lymphoma | 3 yr | Sister | Brain tumour |
| Malignant teratoma of ovary | Sarcoma of liver | 8 yr | Brother | Brain tumour |
| Dysgerminoma of ovary | Ca colon | 14 yr | Mother | Ca brochus |
| ALL[ab] | Osteosarcoma femur | 9 yr | Mother | Ca breast<br>Ca uterus |
| | | | Sister | ALL |
| | | | MGF | Ca colon |
| | | | MU | Glioma |
| | | | MU | Chondrosarc |
| Wilms' tumour | Osteosarcoma rib | 23 yr | Mother<br>Cousin | Ca cervix<br>Wilms' tumour |

[a]Possible Li Fraumeni syndrome families; [b]Sibling with concordant tumour; PGM = Paternal grandmother; MGF = Maternal grandfather; MU = Maternal uncle.

**Table VIII** Treatment factors possibly predisposing to the development of the second tumour in the 151 non simultaneous cases

| *Diagnosis 2nd tumour* | *'RT associated' but no chemo* | *Chemo but not 'RT associated'* | *Chemo & 'RT associated'* | *No chemo & not 'RT associated'* |
|---|---|---|---|---|
| Osteosarcoma (35) | 9 | 6 | 8 | 12 |
| CNS tumour (29) | 13 | 1 | 9 | 6 |
| Carcinoma (22) | 9 | 1 | 2 | 10 |
| Leukaemia (19) | 6 | 3 | 10 | – |
| Skin tumour (18) | 14 | – | – | 4 |
| Soft tissue sarcoma (17) | 3 | 1 | 5 | 8 |
| HD/NHL (4) | – | 2 | 1 | 1 |
| Other (7) | 2 | 1 | – | 4 |
| Total (151) | 56 | 15 | 35 | 45 |

RT associated = Within or on the edge of a radiation field; Chemo = chemotherapy.

**Table IX** Second tumours in patients treated with chemotherapy

| | *Diagnosis of second tumour* | | | | | | | |
|---|---|---|---|---|---|---|---|---|
| *1st tumour* | *Osteo-sarcoma* | *CNS* | *Leukaemia* | *Soft tissue sarcoma* | *Carcinoma* | *Hodgkin's disease* | *Other* | *All* |
| Leukaemia | 1 | 7 | 3 | – | 1 | 3 | | 15 |
| Lymphoma | 2 | 2 | 6 | 2 | – | – | | 12 |
| Retinoblastoma | 8 | 1 | – | 1 | – | – | | 10 |
| Wilms' tumour | 1 | – | – | 3 | 2 | – | | 6 |
| CNS | 1 | – | 2 | – | – | – | | 3 |
| Other | 1 | – | 2 | – | – | – | 1 | 4 |
| Total | 14 | 10 | 13 | 6 | 3 | 3 | 1 | 50 |
| ( )[a] | (40) | (30) | (68) | (33) | (13) | (100) | | |

[a]Figures in brackets represent percentage treated with chemotherapy within that diagnostic group.

**Table X** Chemotherapy given to children developing second tumours

| Drug(s) used | No of cases | No developing leukaemia as 2nd tumour |
|---|---|---|
| Cyclophosphamide as single agent | 10 | – |
| Multiple drugs + cyclophosphamide | 22 | 9 |
| Multiple drugs + other alkylating agent | 6 | 4 |
| Multiple drugs – no alkylating agent | 10 | – |
| Single agent – non alkylating | 2 | – |
| | 50 | 13 |

neoplasia (one with neurofibromatosis and the other with *polyposis coli*).

*Carcinoma* Of the 22 cases of carcinoma developing as a second tumour, 13 were considered to be radiation associated, 5 developed outside radiation portals and 4 had not been irradiated. There were 6 cases of colorectal cancer, 5 of which were radiation associated, and 4 cases of thyroid carcinoma, 3 of which were thought to be related to previous radiotherapy. A difference in the latent intervals to diagnosis of carcinoma was noted between the radiation associated carcinomas, (median interval 14 years) and the remainder (median interval 26 years). There were no obvious dissimilarities between the two groups with regard to factors such as genetic disease, chemotherapy or the spectrum of primary tumour type, to account for the observed difference in the latent interval.

*Leukaemia* Leukaemia developed as a second tumour in 19 patients, at a median interval of 5 years following treatment for the first tumour. Sixteen patients developed a leukaemia classified as either AML or an undifferentiated leukaemia and 3 developed acute lymphoblastic leukaemia. Ten of the children had been treated with both chemotherapy and irradiation, 6 by irradiation alone and 3 with chemotherapy alone (Table XI).

**Table XI** Treatment given to children subsequently developing leukaemia as a second tumour

| Diagnosis 1st tumour | RT alone | Chemo alone | RT + chemo | Total |
|---|---|---|---|---|
| CNS tumour | 3 | – | 2 | 5 |
| Lymphoma (HD/NHL) | – | 2 | 4 | 6 |
| Acute leukaemia | – | – | 3 | 3 |
| Retinoblastoma | 1 | – | – | 1 |
| Osteosarcoma | 1 | – | – | 1 |
| Ewings sarcoma | – | – | 1 | 1 |
| Wilms' tumour | 1 | – | – | 1 |
| Neuroblastoma | – | 1 | – | 1 |
| Total | 6 | 3 | 10 | 19 |

*Skin* Eighteen patients developed a second primary of skin either basal cell carcinoma (14) or malignant melanoma (4); 14 (78%) were considered to be radiation associated. None had received chemotherapy. Three of the 4 patients with malignant melanoma had previously been treated for retinoblastoma and two of the patients with basal cell carcinoma had the basal cell naevus syndrome.

*Soft tissue sarcomas* Five soft tissue sarcomas developed after treatment for a tumour of the CNS, none were radiation associated and none of the patients had received chemotherapy; four occurred in patients with neurofibromatosis. Four soft tissue sarcomas developed after treatment for Wilms' tumour and all were considered to be 'radiation associated'. In addition, three of the four children had been treated with actinomycin-D.

### Discussion

It is evident that the number of children developing second primary tumours is increasing. As many multiple primary tumour cases have already been identified for the decade 1970–79 as for the previous decade, although the period at risk for patients diagnosed during this later period is shorter. This increase may be explained partly by the increased numbers of survivors at risk, although it is likely that treatment factors have contributed to induction of the second malignancy in a significant number of patients. Estimates of the risk of developing a second histologically distinct malignancy following childhood cancer vary substantially. In an analysis of the LESG data by Mike *et al.* (1982), the estimated cumulative risk was 3.3% at 20 years whilst in a study by Li (1977), the cumulative risk was 12% for 5–24 years from diagnosis. For survivors of Ewing's sarcoma, Strong *et al.* (1979) suggested that the cumulative risk of radiation related second tumours might be as high as 35% at 10 years. However since the standard error was 15%, these figures are subject to considerable uncertainty. In a study carried out by the CCRG (Hawkins *et al.*, 1987), about 4% of 3 year survivors of childhood cancer had developed a second primary cancer during the subsequent 20 year period.

The patterns of second tumours appear to be changing; before 1970 the two tumour types most frequently associated with the development of a second tumour were genetic retinoblastoma and tumours of the CNS, whereas since 1970, children with leukaemia and lymphoma have been the major group developing second tumours. This may reflect the improvement in survival for children with leukaemia and lymphoma following the introduction of intensive combined modality treatment programmes and also the longer latent interval for the types of second malignancies most commonly seen in children treated for solid tumours.

The association of acute leukaemia with tumours of the central nervous system (12 patients in this series) has also been noted by Meadows *et al.* (1977) who observed five patients with leukaemia or lymphoma and glioma in an analysis of 102 second malignant neoplasms observed by members of the Late Effects Study Group. Meadows and her colleagues suggested that the association might be part of a new genetic cancer syndrome. Support for this hypothesis comes from a study of 643 children with CNS tumours carried out by Farwell and Flannery, (1984*a*) who found an excess of haemopoietic-lymphatic cancer in the siblings of the children with tumours of the CNS.

In our series, osteosarcoma and tumours of the CNS were the most frequently observed second malignant neoplasms. Twenty one of the osteosarcomas occurred in children with retinoblastoma. Our findings are similar to those of the Late Effects Study Group (Meadows *et al.*, 1985), who have also observed a high frequency of osteosarcoma occurring as a second malignancy in their childhood cancer patients. In our series of 45 children with primary tumours of the CNS, 12 (27%) of the second tumours were also in the CNS. Five of the 12 had evidence of Von Recklinghausen's disease. In the LESG series, (Meadows *et al.*, 1985) there were 31 children who had their first primary in the CNS but of these only 4 (13%) developed a second tumour within the CNS. In a review of 670 children with CNS tumours, Farwell and Flannery (1984*b*) found three children who had developed a second tumour within the CNS; the expected number was 0.16 giving a relative risk of 19.

The main points of difference between the findings of the LESG and those of our study are, firstly, the relative infrequency of children with neuroblastoma developing a second malignancy in our series and, secondly, the relatively larger number of children with CNS tumours and acute leukaemia in our study who have developed second tumours. This may reflect selection in referral patterns within the various institutions of the LESG although it is possible that different therapeutic approaches may have contributed to the differences observed. Also the early LESG reports included only patients diagnosed before 1970, when few cases of leukaemia survived.

In this series, a total of 16 children with acute leukaemia, 14 with ALL and two children with AML, developed second tumours, a larger number than has been reported in any other single series. Thirteen developed a solid tumour (CNS (7), Hodgkin's disease (3), osteosarcoma (1), neuroblastoma (1) and carcinoma of parotid gland (1)) and three a new leukaemia. Askold *et al.* (1981) reviewed the literature and found reports of 33 children with ALL who had developed a second malignancy. Nine of these had developed a solid tumour and 13 a second leukaemia. Seven of the cases had developed histiocytic medullary reticulosis (HMR) as the second malignancy at intervals of 3–8 months following the diagnosis of ALL. Although we know of two such cases in Britain we have not included them in our series because of the doubt about the pathogenesis of HMR. There have been reports suggesting that HMR is a reaction to a viral infection in an immunocompromised host (Risdall *et al.*, 1979).

In this series of 151 patients with non-simultaneous tumours, 77 of the 125 children treated with radiation developed their second tumour within or on the edge of the radiation field whilst a further 16 children developed acute leukaemia making a total of 93 (61%) which could be described as 'radiation associated'. Twenty one of these radiation related tumours occurred in children who also had a genetically determined susceptibility. In a report by Li and colleagues (1977), fifteen of 410 patients surviving for 5 years or more developed a second malignant tumour and all but one of the fifteen second cancers described arose in tissues previously irradiated. In an analysis of the LESG data (Meadows *et al.*, 1985), 208 (67%) of 308 second or subsequent tumours were classified as radiation associated. The carcinogenic potential of low doses of radiation in the development of tumours such as thyroid, salivary gland and brain tumours has also been stressed (Modan *et al.*, 1974; Curtin *et al.*, 1977). Twenty five of the second tumours associated with radiation therapy in this series developed on the edge of a radiation field.

The association between retinoblastoma and osteosarcomas occurring either within or outside the radiation field is well recognised (Reese *et al.*, 1949; Abramson *et al.*, 1976). In addition, there appears to be a specific association between retinoblastoma and other types of sarcoma and possibly also with melanoma (DerKinderen *et al.*, 1986). In this series there were 21 cases of osteosarcoma following treatment for retinoblastoma. Equal numbers of the osteosarcomas developed in long bones outside the radiation field as developed within the radiation field, and a further five had not received any radiation. This suggests that genetic predisposition is probably the underlying factor in the development of second tumours in patients with retinoblastoma; whilst the development of osteosarcomas in bones of the orbit, an extremely rare site for primary osteosarcoma, suggests that patients with genetic retinoblastoma may demonstrate a radiation sensitivity. The evidence for these conclusions and a more detailed analysis of the second primary tumours occurring in our series of children with retinoblastoma are presented elsewhere (Draper *et al.*, 1986).

Fifty children in our series had received cytotoxic drugs. A number of chemotherapeutic agents have been implicated in the development of second malignancies (Schmahl *et al.*, 1982) and the drugs most frequently reported have been the alkylating agents including busulphan, chlorambucil, melphalan and cyclophosphamide. Thirty eight children in this series had received an alkylating agent and in 32 this was cyclophosphamide.

Since the introduction of intensive chemotherapy during the early 1970s, the second malignancy most frequently observed in association with cytotoxic therapy has been acute nonlymphocytic leukaemia (ANLL). In this series, 13 of the 38 second neoplasms that developed after treatment with an alkylating agent were acute leukaemias. In an analysis of the LESG data (Meadows *et al.*, 1985), 49 of 292 children developing second tumours had been treated with chemotherapy alone and all but two had been treated with at least one alkylating agent. Twelve of the 49 cases were secondary leukaemias. A significantly increased risk of secondary leukaemia has been shown in a separate analysis of two year survivors of childhood cancer treated in institutions of the LESG (Tucker *et al.*, 1984). The relative risk of secondary leukaemia was strongly associated with the dose of alkylating agent. Several authors have suggested that the risk of developing a second malignant neoplasm is greatest in patients receiving both chemotherapy and radiotherapy, and there is considerable evidence for this from studies of adult patients (Cadman *et al.*, 1971; Arseneau *et al.*, 1977). Forty five children (30%), in this series had received both chemotherapy and radiotherapy although in only 35 of these was the radiotherapy thought to have been contributory to the induction of the second tumour.

The occurrence of tumours of the uterus in two patients following oestrogen replacement therapy is of particular interest in view of the occurrence of endometrial cancer following the use of oestrogens in post menopausal women (Jick *et al.*, 1980). The development of an osteosarcoma in a child with a pituitary adenoma treated with growth hormone is also noteworthy. Meadows and colleagues, (1980), reported that the development of bone sarcoma occurring as a second neoplasm was significantly influenced by prior radiation therapy and genetic predisposition. In a subsequent analysis of the LESG data, Tucker and colleagues (1985), showed that exposure to alkylating agents was associated with a significant 2-fold risk of bone cancer and that this was independent of radiation therapy. There is a high incidence of osteosarcoma during adolescence with the peak occurring earlier in girls than in boys, possibly related to the earlier growth spurt in girls. As the normal growth spurt is under hormonal control it is interesting to speculate that an abnormal hormonal influence might also be a factor in the development of secondary osteosarcoma.

It is well recognised that genetic factors can influence the development of a malignancy and in addition to the genetic form of retinoblastoma, several inherited conditions such as the basal cell naevus syndrome, Von Recklinghausen's disease and tuberose sclerosis are known to be associated with the development of childhood cancer and of multiple primary neoplasms (Mulvihill 1977). Meadows *et al.* (1985) found the influence of genetic or familial factors in 82 of 292 (28%), of their patients and 73 (25%) had a genetic disease known to predispose to cancer, a much higher incidence than in the overall population of children who develop cancer. A recognised genetic disease was present in 53 (33%) of our total series of 161 cases and there was a family history of malignancy in at least one first degree relative in a further 8 cases.

Cancers that have a genetic basis often occur as multiple tumours which are relatively tissue specific, for example osteosarcoma with bilateral retinoblastoma. The retinoblastoma gene has been mapped to band 14 on the long arm of chromosome 13 and mutations at this locus result in the development of retinoblastoma. Of considerable interest is some recent research work which has demonstrated that osteosarcoma may result from a similar mutation at the q14 band of chromosome 13 (Dryja *et al.*, 1986). Observation of the patterns of double tumours may help direct paths of research in molecular biology and thereby increase our understanding of the molecular mechanisms predisposing to the development of multiple primary tumours.

In conclusion, the number of second primary tumours can be expected to increase as a consequence of the growing numbers of long term survivors of childhood cancer and as the trend for more intensive chemotherapy and combined modality treatments becomes more widely accepted. We predict that this increase will be most pronounced in those childhood cancer survivors with a genetic susceptibility. Therefore it behoves clinicians to identify children with cancer prone conditions so that treatment protocols containing potentially oncogenic therapies such as alkylating agents and irradiation may be reconsidered for such patients. With the introduction of new, effective, non-alkylating cytotoxic agents, it may be possible to eliminate alkylating agents from protocols for patients with a favourable prognosis, thereby reducing the risk of inducing a second tumour without compromising survival. Long term follow up of childhood cancer patients is needed to determine the magnitude of the problem of second primary malignancies and to continue identification of possible aetiological factors.

We are grateful to Professor D.G. Harnden, Professor J.S. Malpas, Dr J.R. Mann, Dr P.H. Morris Jones and Dr D. Pearson, members of the working party who advised and helped establish this study. We thank the many consultants and general practitioners who provided the material on which this paper is based. We are grateful to the Office of Population Censuses and Surveys, the Information Services Division of the Common Services Agency of the Scottish Health Service, the Registrar General of Scotland and regional cancer registries for providing copies of notifications of childhood cancer cases. We thank the National Health Service Central Registers at Southport and Edinburgh for notification of deaths and 'flagging' of survivors.

The Childhood Cancer Research Group is supported by the Department of Health and Social Security and the Scottish Home and Health Department. The Long term Follow-up study of childhood cancer survivors on which this study is based is supported by the Cancer Research Campaign and the Leukaemia Research Fund.

### References

ABRAMSON, D.H., ELLSWORTH, R.M. & ZIMMERMAN, L.E. (1976). Non-ocular cancer in retinoblastoma survivors. *Trans. Am. Acad. Opthalmol. Otolaryngol.*, **81**, 454.

ABRAMSON, D.H., ELLSWORTH, R.M., KITCHIN, F.D. & TUNG, G. (1984). Second nonocular tumors in retinoblastoma survivors. Are they radiation induced. *Ophthalmology*, **91**, 1351.

ANDERSON, J.R. & TREIP, C.S. (1984). Radiation-induced intracranial neoplasms. A report of three possible cases. *Cancer*, **53**, 426.

ARSENEAU, J.C., CANELLOS, G.P., JOHNSON, R. & DE VITA, V.T. (1977). Risk of new cancers in patients with Hodgkin's disease. *Cancer*, **40**, 1912.

ASKOLD, L.T.C., MOSIJCZVK, A.D. & RUYMANN, F.B. (1981). Second malignancy in acute lymphoblastic leukaemia. Review of 33 cases. *Am. J. Dis. Child.*, **92**, 313.

BONNIN, J.M., RUBINSTEIN, L.J., PALMER, N.F. & BECKWITH, J.B. (1984). The association of embryonal tumors originating in the kidney and in the brain: A report of seven cases. *Cancer*, **54**, 2137.

CADMAN, E.C., CAPIZZI, R.L. & BERTINO, J.R. (1971). A.N.L.L. A delayed complication of Hodgkin's disease therapy. *Cancer*, **40**, 1280.

CURTIN, C.T., MCHEFFY, B. & KOLARSICK, A.J. (1977). Thyroid and breast cancer following childhood radiation. *Cancer*, **40**, 2911.

DER KINDEREN, D.J., KOTEN, J.W., DEN OTTER, W., TAN, K.E.W.P. & BEEMER, F.A. (1986). Retinoblastoma, melanoma, and pancreatic cancer. *Lancet*, **ii**, 1335.

DRAPER, G.J., BIRCH, J.M., BITHELL, J.F. & 6 others (1982). Childhood Cancer in Britain. Incidence, survival and mortality. *Studies on Medical and Population subjects*, 37, HMSO.

DRAPER, G.J., SANDERS, B.M. & KINGSTON, J.E. (1986). Second primary neoplasms in patients with retinoblastoma. *Br. J. Cancer*, **53**, 661.

DRYJA, T., RAPAPORT, J., EPSTEIN, J. & 4 others (1986). Chromosome 13 homozygosity in osteosarcoma without retinoblastoma. *Am. J. Hum. Genet.*, **38**, 59.

FARWELL, J. & FLANNERY, J.T. (1984). Cancer in relatives of children with central-nervous-system neoplasms. *N. Engl. J. Med.*, **311**, 749.

FARWELL & FLANNERY, J.T. (1984). Second primaries in children with central nervous system tumours. *J. Neuro-Oncol.*, **2**, 371.

HAWKINS, M.M., DRAPER, G.J. & KINGSTON, J.E. (1987). Incidence of second primary tumours among childhood cancer survivors. *Br. J. Cancer*, **56**, 339.

INGRAM, L., MOTT, M.G., MANN, J.R., RAAFAT, F., DARBYSHIRE, P.J. & MORRIS JONES, P.H. (1987). Second malignancies in children treated for non-Hodgkin's lymphoma and T-cell leukaemia with the UKCCSG regimens. *Br. J. Cancer*, **55**, 463.

JICK, H., WALKER, A.M. & ROTHMAN, K.J. (1980). The epidemic of endometrial cancer. A commentary. *Am. J. Public Health*, **20**, 264.

JUDGE, M.R., EDEN, O.B. & O'NEILL, P. (1984). Cerebral glioma after cranial prophylaxis for acute lymphoblastic leukaemia. *Br. Med. J.*, **289**, 1038.

KINGSTON, J.E., PLOWMAN, P.N. & HUNGERFORD, J.L. (1985). Ectopic intracranial retinoblastoma in childhood. *Br. J. Ophthalmol.*, **69**, 742.

KORIECH, O.M. & MCNAUGHT, G.H.D. (1981). Second primary neoplasm in a dysgerminoma patient. *Br. J. Radiol.*, **54**, 1005.

LEE, W.R., LAURIE, J. & TOWNSEND, A.L. (1975). Fine structure of a radiation induced osteosarcoma. *Cancer*, **36**, 1414.

LI, F.P. (1977). Second malignant tumours after cancer in childhood. *Cancer*, **40**, 1899.

LI, F.P. & FRAUMENI, J.F. (1969). Soft tissue sarcomas, breast cancer and other neoplasms. *Ann. Intern. Med.*, **71**, 747.

MEADOWS, A.T., D'ANGIO, G.J., MIKE, V. & 4 others (1977). Patterns of second malignant tumours in children. *Cancer*, **40**, 1903.

MEADOWS, A.T., STRONG, L.C., LI, F. & 6 others (1980). Bone sarcoma as a second malignant neoplasm in children: Influence of radiation and genetic predisposition. *Cancer*, **46**, 2603.

MEADOWS, A.T., BAUM, E., FOSSATI-BELLANI, F. & 10 others (1985). Second malignant neoplasms in children: An update from the Late Effects Study Group. *J. Clin. Oncol.*, **3**, 532.

MIKE, V., MEADOWS, A.T. & D'ANGIO, G.J. (1982). Incidence of second malignant neoplasms in children: Results of an international study. *Lancet*, **ii**, 1326.

MODAN, B., BAIDATZ, D., MART, H., STEINITZ, R. & LEVIN, S.G. (1974). Radiation induced head and neck tumours. *Lancet*, **i**, 277.

MULVIHILL, J.J. (1977). Genetics of multiple primary tumours. *Cancer*, **40**, 1867.

PEARSON, A.D.J., CRAFT, A.W., PERRY, R.H., KALBAG, R.M. & EVANS, R.G.B. (1983). Four primary tumours in one child. *Cancer*, **52**, 2363.

PRENTICE, A.G., SMITH, A.G. & BRADSTOCK, K.F. (1980). Mixed lymphoblastic-myelomonoblastic leukaemia in treated Hodgkin's disease. *Blood*, **56**, 129.

REESE, A.B., MERRIAM, G.R. & MARTIN, H.E (1949). Treatment of bilateral retinoblastoma by irradiation and surgery. *Am. J. Ophthalmol.*, **32**, 175.

RISDALL, R.J., MCKENNA, R.W., NESBIT, M.E. & 3 others (1979). Virus associated hemophagocytic syndrome. A benign histiocytic proliferation distinct from malignant histiocytosis. *Cancer*, **44**, 993.

SCHMAHL, D., HAB, M., LORENZ, M. & WAGNER, I. (1982). Occurence of second tumours in man after anti cancer drug treatment. *Cancer Treatment Rev.*, **9**, 167.

SECKER-WALKER, L.M., STEWART, E.L. & TODD, A. (1985). Acute lymphoblastic leukaemia with t(4;11) follows neuroblastoma: A late effect of treatment? *Med. Pediatr. Oncol.*, **13**, 48.

STEVENSON, J.C., SPANOS, E. & ACKROYD, N. (1981). Sipple syndrome: Marked variability of the disease within a family and implications for management. *Postgrad. Med. J.*, **57**, 104.

STRONG, L.C., HERSON, J., OSBORNE, B.M. & SUTOW, W.W. (1979). Risk of radiation related subsequent malignant tumours in survivors of Ewing's sarcoma. *J. Natl Cancer Inst.*, **62**, 1401.

TUCKER, M.A., MEADOWS, A.T., BOICE, J.D. *et al* for the Late Effects Study Group (LESG). (1984). Secondary leukaemia (SL) after alkylating agents (AA) for childhood cancer. *Am. Soc. Clin. Oncol*, C-332 (*Abstract*).

TUCKER, M.A., MEADOWS, A.T., BOICE, J.D. *et al* for the Late Effects Study Group (LESG). (1985). Bone cancer (BC) linked to radiotherapy and chemotherapy in children. *Am. Soc. Clin. Oncol.*, C-932 (*Abstract*).

WARREN, S. & GATES, O. (1932). Multiple primary malignant tumours. *Am. J. Cancer*, **16**, 1358.